# Efficient Turn-On Fluorescent Sensor Based on Fluorescent Resonance Energy Transfer between 1,3,6,8-Tetra(4-pyridyl)pyrene and Gold Nanoparticles for Glutathione Detection

**DOI:** 10.1021/acsomega.5c05251

**Published:** 2025-10-16

**Authors:** Shicong Liu, Yuanyuan Zhang, Dan Jia, Junqiu Liu, Chunxi Hou

**Affiliations:** State Key Laboratory of Supramolecular Structure and Materials, College of Chemistry, Jilin University, 2699 Qianjin Street, Changchun 130012, China

## Abstract

A fluorescent probe based on fluorescent resonance energy transfer (FRET) between the organic fluorophore 1,3,6,8-tetra­(4-pyridyl) pyrene (TTPY) and gold nanoparticles (AuNPs) was designed for detecting glutathione (GSH) in foods. TTPY, synthesized via a Suzuki–Miyaura coupling reaction, exhibited excellent fluorescence properties with a maximum emission wavelength of 496 nm. Acting as an energy donor, TTPY transfers energy to AuNPs via FRET, resulting in fluorescence quenching of TTPY. In the presence of GSH, TTPY was displaced from the AuNP surface, restoring the fluorescence of the TTPY chromophore. The developed sensor demonstrated excellent water solubility and selectivity and a low detection limit (54 nM). Moreover, the proposed method was successfully applied for the determination of GSH in food samples, demonstrating its potential for food antioxidant analysis.

## Graphical abstract

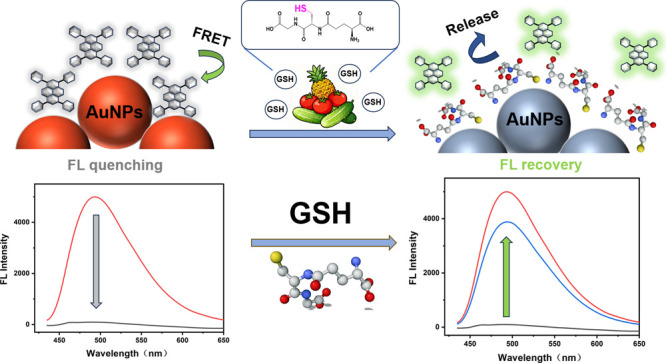

## 1 Introduction

Glutathione (GSH), the most abundant intracellular nonprotein thiol and an essential antioxidant, plays a pivotal role in maintaining diverse physiological functions including protecting cells from oxidative damage, preserving cellular redox homeostasis, exhibiting anticarcinogenic effects, and delaying aging processes.^1–4^ Fresh fruits and vegetables are recognized as important dietary sources of GSH for the human body.^5–8^ Abnormal levels of GSH in humans serve as a biomarker for various diseases, including Parkinson’s disease, hepatic injury, Alzheimer’s disease, and neurodegenerative disorders.^9–12^ Therefore, the development of an effective and cost-effective method for detecting GSH contents in food is significant. To date, numerous techniques have been used for GSH detection including high-performance liquid chromatography,^13–15^ electrochemical methods,^16,17^ surface-enhanced Raman scattering,^18–20^ colorimetry,^21–23^ mass spectrometry,^24,25^ capillary electrophoresis,^26^ and enzyme-linked immunosorbent assay.^27,28^ However, these methods often suffer from limitations such as high cost, and complicated and time-consuming operation procedures. Recently, fluorescence-based detection has gained widespread attention owing to its rapid response, high sensitivity, simple operation, low cost, and real-time monitoring capability.^29,30^

Gold nanoparticles (AuNPs) have emerged as attractive materials for sensing various substances owing to their unique size-dependent optical and electronic properties.^31,32^ Their high molar extinction coefficient (up to 10^8^ M^–1^ cm^–1^) and broad absorption spectrum enable AuNPs to act as ultraefficient fluorescence quenchers through the fluorescent resonance energy transfer (FRET) process between fluorophores and AuNPs.^33,34^ Leveraging this property of AuNPs, several fluorescence-based methods utilizing AuNP conjugates have been developed to detect various analytes. Zhang and co-workers fabricated a water-soluble nanosensor based on FRET between AuNPs and the organic fluorophore (2-butyl-6-pyridin-4-yl-benzo­[de] isoquinoline-1,3-dione), which could rapidly sense biothiols^35^ in living cells. Dong and co-workers designed a novel turn-on fluorescence biosensor for GSH detection based on a FRET system comprising nitrogen (N) and sulfur (s) codoped carbon dots (N,S-CDs) and AuNPs.^36^ Negatively charged AuNPs bind to N,S-CDs via electrostatic interactions, inducing FRET-induced fluorescence quenching of N,S-CDs. The addition of GSH restored the fluorescence of N,S-CDs, with the degree of fluorescence recovery exhibiting a linear correlation with the GSH concentration.

Pyrene, a widely used organic fluorophore with an extensive conjugated structure, exhibits excellent fluorescence properties and chemical stability.^37,38^ Leveraging the unique architecture of pyrene, its derivatives have been extensively used as fluorescent probes in photophysical research. Kathiravan and co-workers developed a pyrene-tethered 1-(pyridin-2-yl)­imidazo­[1,5-*a*]­pyridine-based fluorescent probe, which functioned as a fluorescent chemical sensor for nitro derivative–based explosives, which selectively and sensitively detected picric acid.^39^ However, pyrene derivatives have rarely been used for the detection of biothiols. Therefore, herein, we designed and synthesized 1,3,6,8-tetra­(4-pyridyl)­pyrene (TTPY) and designed a water-soluble nanosensor based on FRET between AuNPs and TTPY (AuNP–TTPY) to rapidly sense biothiols (Scheme 1). Owing to the synergistic effect of electrostatic interactions and weak N–Au interactions, TTPY coordinates to the surface of AuNPs, resulting in efficient fluorescence emission quenching of TTPY. In the presence of GSH, the TTPY chromophore was displaced from the AuNP surface owing to the strong Au–thiol affinity, resulting in a significant fluorescence recovery. The recovery degree of fluorescence intensity was linearly related to the GSH concentration. Based on this strategy, an AuNP-based sensor was developed for detecting thiols in aqueous solution and real samples.

**Scheme 1:**
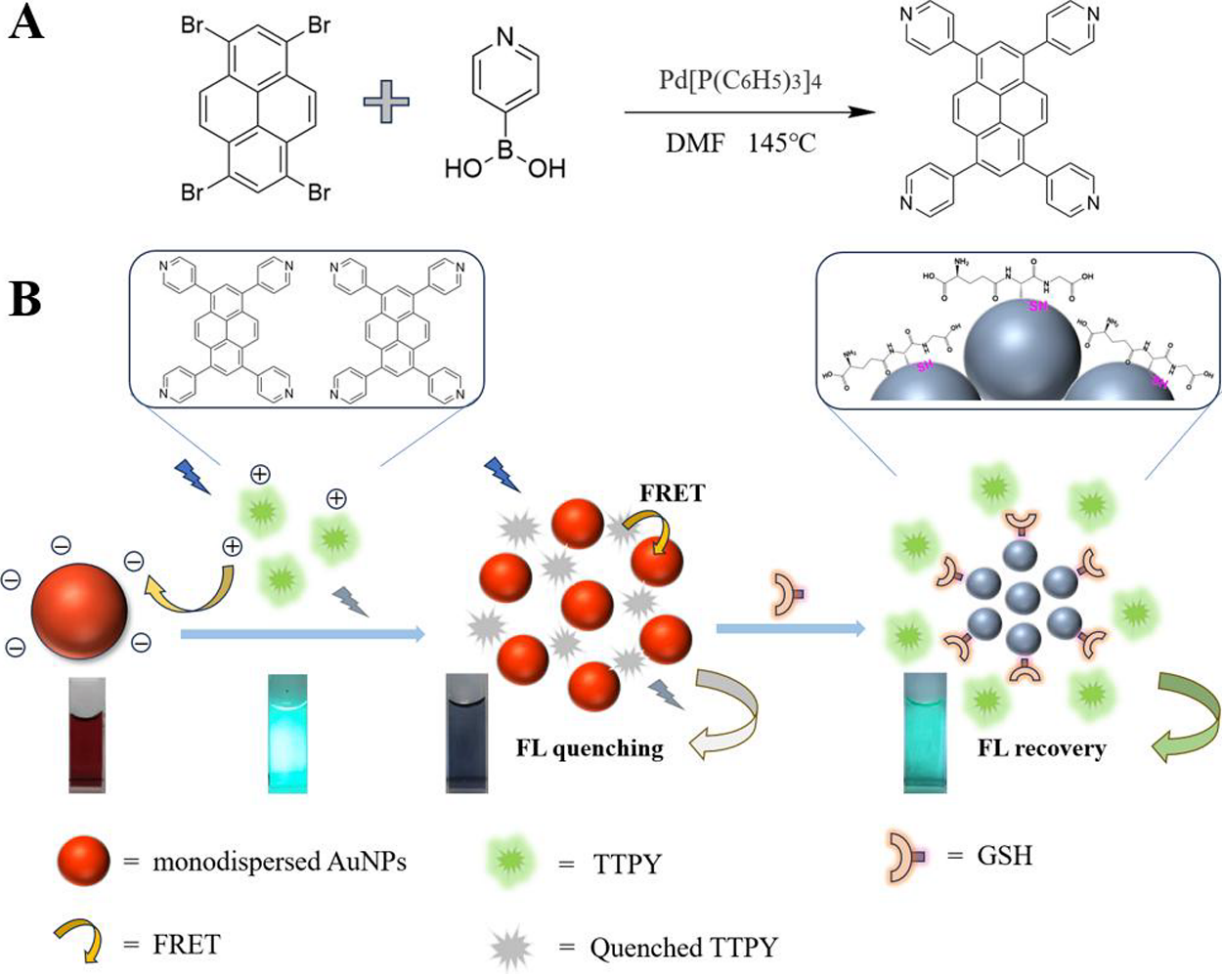
(A) Synthesis of 1,3,6,8-Tetra­(4-pyridyl)­pyrene; (B) Schematic Illustration of the Fluorescence-Based GSH Detection Using AuNP-TTPY

## 2 Experimental Section

### 2.1 Chemicals

Chloroauric acid trihydrate (HAuCl_4_·3H_2_O), trisodium citrate (99.8%), GSH, 1,3,6,8-tetrabromopyrene, 4-pyridylboronic acid, *N*,*N*-dimethylformamide (DMF), potassium carbonate (K_2_CO_3_), tetrakis­(triphenylphosphine)­palladium(0) [Pd­(PPh_3_)_4_], anhydrous acetic acid (CH_3_COOH) were purchased from Energy Chemical and Aladdin Reagent Company without further purification unless otherwise specified. All glassware used in the experiments was cleaned using aqua regia to avoid any possible contamination, followed by rinsing with ultrapure water, and was dried before use.

### 2.2 Apparatus and Characterization

Fluorescence spectra were recorded using an RF-5301PC fluorescence spectrophotometer (Shimadzu Co., Japan). Ultraviolet–visible (UV–vis) absorption spectra were acquired using a SHIMADZU2450 UV–vis spectrophotometer (Shimadzu Co., Japan). High-resolution transmission electron microscopy (HRTEM, JEM-2100F, Japan Electron Optics Laboratory Co., Ltd.) was used to characterize the morphology of the synthesized nanomaterials. Dynamic light scattering (DLS) measurements were performed using a Zetasizer Nano ZSE device (Malvern Instruments Ltd., UK). Fourier transform infrared (FTIR) spectra were obtained using a VERTEX 80 V FTIR spectrometer (Nicolet Instrument Co., USA).

### 2.3 Synthesis of TTPY

1,3,6,8-Tetrabromopyrene (1.04 g, 2 mmol) was added to a reaction flask, followed by 983 mg (8 mmol) of 4-pyridylboronic acid, 200 mL of DMF, and 0.42 g of K_2_CO_3_. The mixture was degassed by bubbling nitrogen through it for 30 min. Subsequently, Pd­(PPh_3_)_4_ (0.138 g, 0.12 mmol) was added, followed by further degassing for 10 min. The reaction mixture was heated to 145 °C and maintained at this temperature for 48 h. Upon cooling, the mixture was poured into 1 L of water and stirred vigorously for 30 min to facilitate precipitation. The resulting precipitate was collected via filtration and washed sequentially with 100 mL portions of water, methanol, and dichloromethane. The obtained product was a green solid (851 mg, 1.66 mmol, 83%) (500 MHz, acetic acid-d4): δ (ppm) = 9.10 (d, *J* = 6.0 Hz, 8H), 8.43 (s, 4H), 8.36 (s, 2H), 8.17 (d, *J* = 6.0 Hz, 8H).

### 2.4 Preparation of the AuNP–TTPY Composite

A round-bottom flask was filled with 9.75 mL of deionized water, followed by the addition of 250 μL of 40 mM HAuCl_4_·3H_2_O. The mixture was refluxed under vigorous stirring. Thereafter, 1 mL of 39.1 mM trisodium citrate dihydrate was rapidly added to the boiling solution, and the solution immediately changed from light yellow to gray and gradually deepened to deep wine red over the next few minutes. The mixture was refluxed for a further 15 min to ensure the complete formation of nanoparticles. The colloidal solution was allowed to cool to room temperature. The AuNP dispersion was filtered through a 0.45-μm membrane filter to eliminate aggregates. The purified AuNPs were stored at 4 °C in the dark to prevent degradation.

TTPY (5 μL, 1 mM) was added to 1 mL of AuNP solution in the dark. The resulting mixture was stirred for 10 min at room temperature. The obtained AuNPs–TTPY solution was diluted with 10 mmol L^–1^ phosphate-buffered saline buffer (pH = 4.0) to obtain the stock solution.

### 2.5 General Procedure for the Detection of GSH

According to fluorescence titration conditions, the corresponding molar ratios of GSH were added to the AuNP–TTPY solution ([TTPY] = 1.3 μM and [AuNPs] = 1.2 nM). After stirring for 9 min, the fluorescence was measured at room temperature.

### 2.6 Detection of GSH in Actual Samples

In actual sample testing, fruit samples were first washed and peeled. The juice was extracted and homogenized using a blender. The processed juice was centrifuged at 4000 rpm for 10 min, and the supernatant was filtered through a 0.45-μm membrane filter.^40^ The filtrate was diluted with ultrapure water to an appropriate concentration within the detection range for spectroscopic analysis. Fruit samples spiked with known concentrations of GSH underwent the same pretreatment procedure.

## 3 Results and Discussion

### 3.1 Characterization of TTPY, AuNPs, and AuNP–TTPY

The optical properties and surface chemical structure of the synthesized nanomaterials were analyzed via UV–vis absorption, fluorescence, and FTIR spectroscopy, HRTEM, and DLS. Figure 1A shows that TTPY exhibits a broad absorption band with an absorption maximum of 417 nm. Figure 1A (inset) shows the bright blue–green fluorescence of TTPY in an aqueous solution under a 365 nm UV lamp. The fluorescence spectra of TTPY reveal its excellent optical performance in aqueous media, exhibiting a strong emission peak at a wavelength (λ) of 496 nm and an excitation peak at a λ of 417 nm. TTPY coordinates with AuNPs through nitrogen atoms present in its pyridyl groups. The successful formation of TTPY and the AuNP–TTPY complex was confirmed via FTIR spectroscopy results. Figure 1B shows the FTIR spectra of TTPY, AuNP–TTPY, and AuNPs. The broad absorption peak at 3436 cm^–1^ is attributed to O–H stretching vibrations. The two peaks at 2926 and 2850 cm^–1^ are assigned to C–H stretching vibrations. The peak at 1650 cm^–1^ is attributed to C=O stretching vibrations. A new peak at 1029 cm^–1^, corresponding to pyridyl ring breathing vibration compared with the original peak of free pyridyl groups at 1010 cm^–1^indicates coordination between the pyridyl groups and AuNP surface. A comparison of the IR results with the red shift in the UV–vis spectra of AuNP–TTPY with bare AuNPs (Figures S1–S3) confirms the successful conjugation between AuNPs and TTPY.

**Figure 1:**
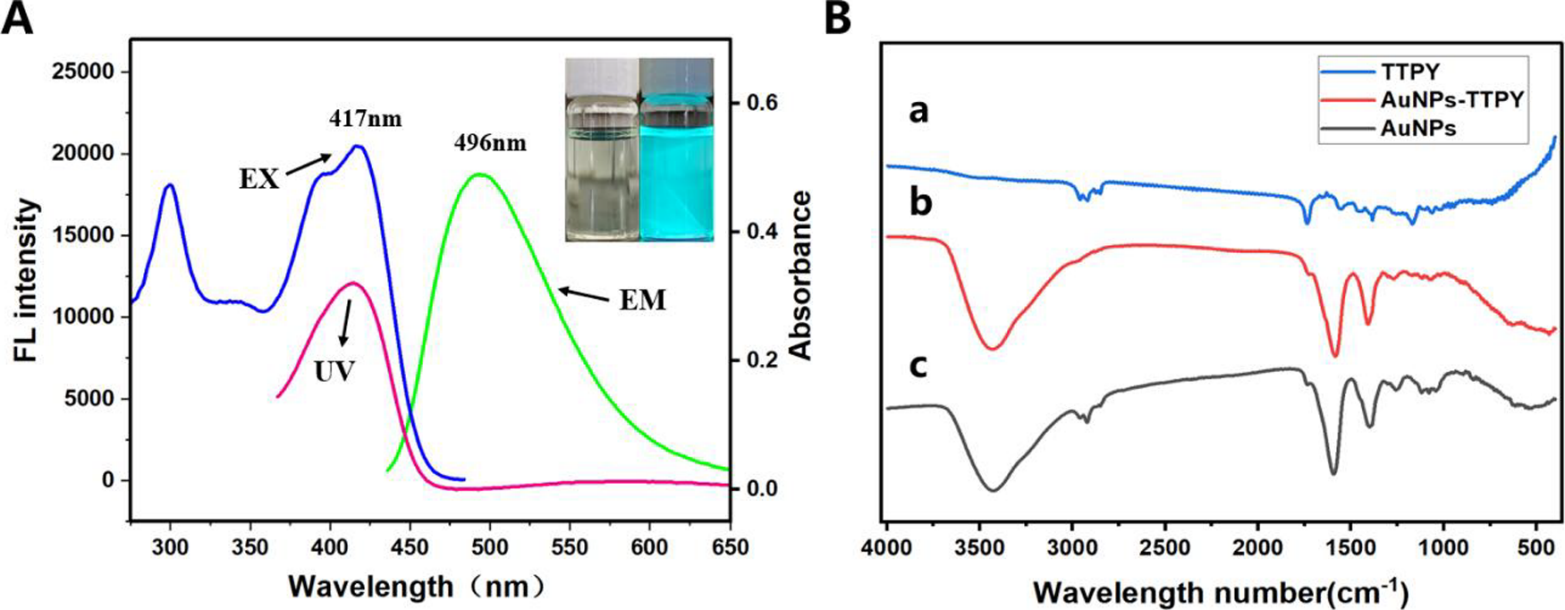
(A) UV–vis absorbance (red), excitation (blue), and emission (green) spectra of TTPY. Inset: image of TTPY under natural light and 365 nm UV light. (B) FTIR spectra of (a) TTPY, (b) AuNP–TTPY, and (c) AuNPs.

The prepared AuNPs and AuNP–TTPY were further characterized via TEM and DLS. Figure 2A shows the TEM images of AuNPs, which are monodispersed, uniform spheres with an average size of ∼13.8 nm. The TEM images of AuNP–TTPY indicate that it comprises spherical nanoparticles with an average size of ∼14.1 nm, with no significant difference in size distribution (Figure 2B). After the addition of GSH, AuNPs maintain a uniform distribution, indicating that their morphology remains unchanged during the thiol-triggered ligand exchange process (Figure 2C). The DLS data indicate average hydrodynamic diameters of AuNPs, AuNP–TTPY, and GSH-added AuNPs are 14.81,14.90, and 15.21 nm, respectively (Figure 2D–F), with a narrow size distribution and well-defined particle sizes of nanoparticles. The sample dimensions observed via TEM are smaller than those determined via DLS owing to structural collapse during the drying process. DLS measures the hydrodynamic diameter in solution, encompassing the nanoparticle core and its hydration layer, whereas TEM captures the dried state with collapsed surface structures.

**Figure 2:**
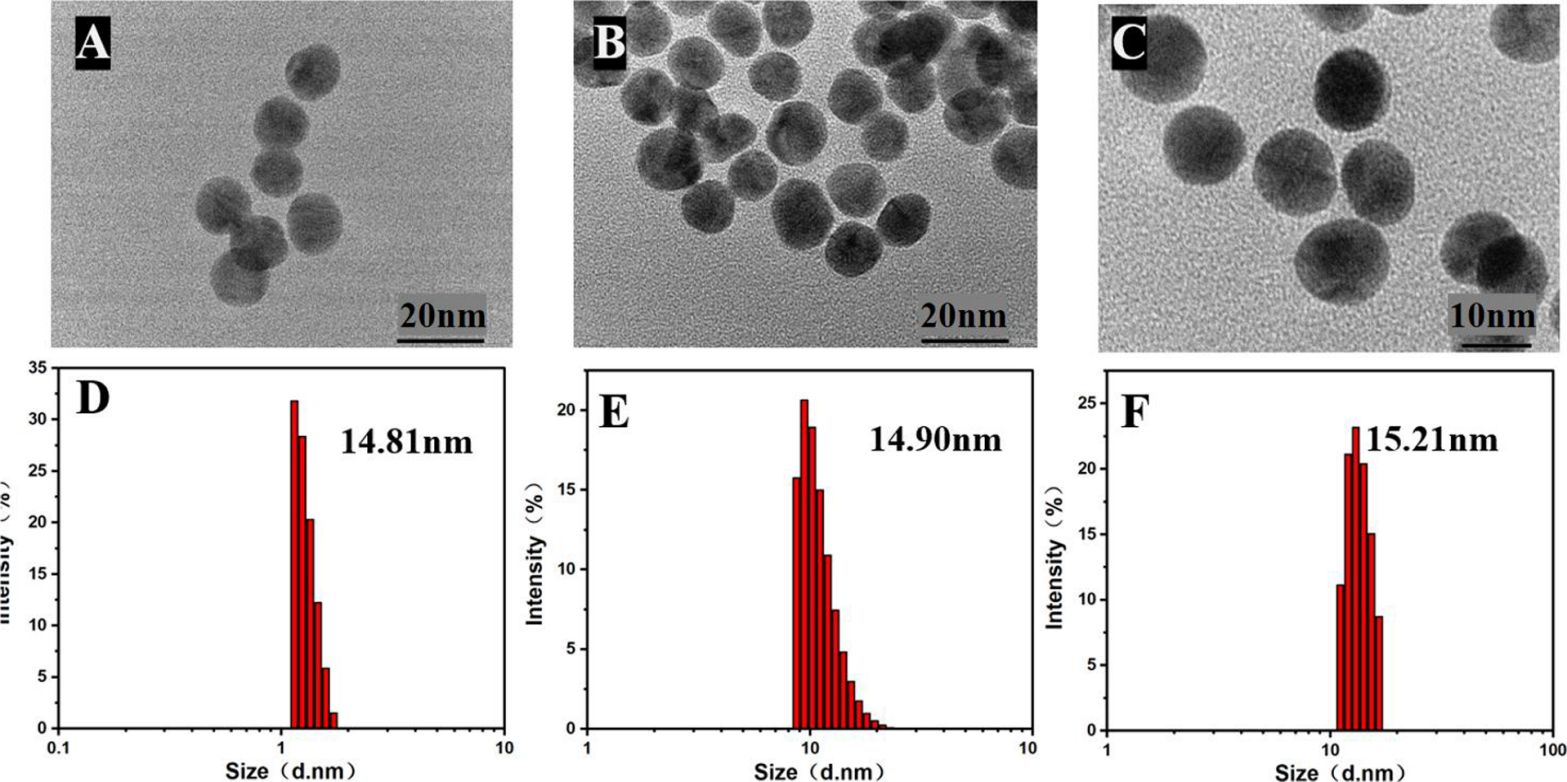
TEM images: (A) monodispersed AuNPs, (B) AuNPs with TTPY, and (C) AuNPs with TTPY and GSH. DLS measurements: (D) monodispersed AuNPs, (E) AuNPs with TTPY, and (F) AuNPs with TTPY and GSH.

### 3.2 FRET System Design between TTPY and AuNPs

Pyrene and its derivatives are widely used as fluorescent probes owing to their excellent fluorescence, low toxicity, and ease of chemical modification.^41^ Scheme 1A outlines the synthesis procedure of the probe TTPY. Pd­(PPh_3_)_4_ catalyzed the reaction of 1,3,6,8-tetrabromopyrene with 4-pyridylboronic acid in degassed anhydrous DMF to afford a green solid [1,3,6,8-tetra­(4-pyridyl)­pyrene] with 83% yield. The detection principle is presented in Scheme 1B. Anhydrous acetic acid–treated TTPY is water-soluble and positively charged in aqueous solution (Figures S3 and S4). TTPY functions as a fluorescent sensor, with its pyridyl groups acting as binding sites for AuNPs. In the absence of thiols, TTPY coordinates with AuNPs via weak N–Au and electrostatic interactions, causing fluorescence quenching of the chromophore through efficient FRET. The sensing mechanism relies on the competitive displacement of TTPY by GSH on AuNPs surfaces, driven by the distinct binding thermodynamics: the Au–thiol bond (*K*_a_ ≈ 10^7^ M^–1^) in GSH is significantly stronger than the N–Au coordination (*K*_a_ ≈ 10^3^ M^–1^) in TTPY. In the presence of thiols, TTPY is displaced from AuNPs owing to the stronger coordination capability of thiols with AuNPs than that with pyridyl groups. Consequently, the released TTPY restores the fluorescence of the sensing system, producing a turn-on signal response.

Figure 3A shows a characteristic plasmonic peak of AuNPs at 520 nm.^42^ The spectral overlap between the emission wavelength of TTPY and the absorption wavelength of AuNPs enables effective FRET with TTPY acting as the donor and AuNPs as the acceptor.^43^ To further verify the FRET effect between AuNPs and TTPY, the fluorescence lifetimes (Figure S5) and quantum yields (Figure S6) of TTPY and AuNPs-TTPY were determined. The results show that the fluorescence lifetime of TTPY is 3.69 ns. Upon conjugation with AuNPs and the occurrence of FRET, the fluorescence lifetime decreases to 3.40 ns. The quantum yield of TTPY is 58.13%. Following fluorescence quenching of TTPY by AuNPs, the quantum yield decreases to 1.87%. As expected, AuNP–TTPY exhibits negligible fluorescence emission (Figure 3B). The addition of 10-μM GSH to AuNP–TTPY exhibits a sharp increase in fluorescence intensity at 496 nm, accompanied by intense green fluorescence emission under UV light. This 40-fold enhancement in fluorescence intensity demonstrates this system as a highly sensitive thiol-sensing platform to date.

**Figure 3:**
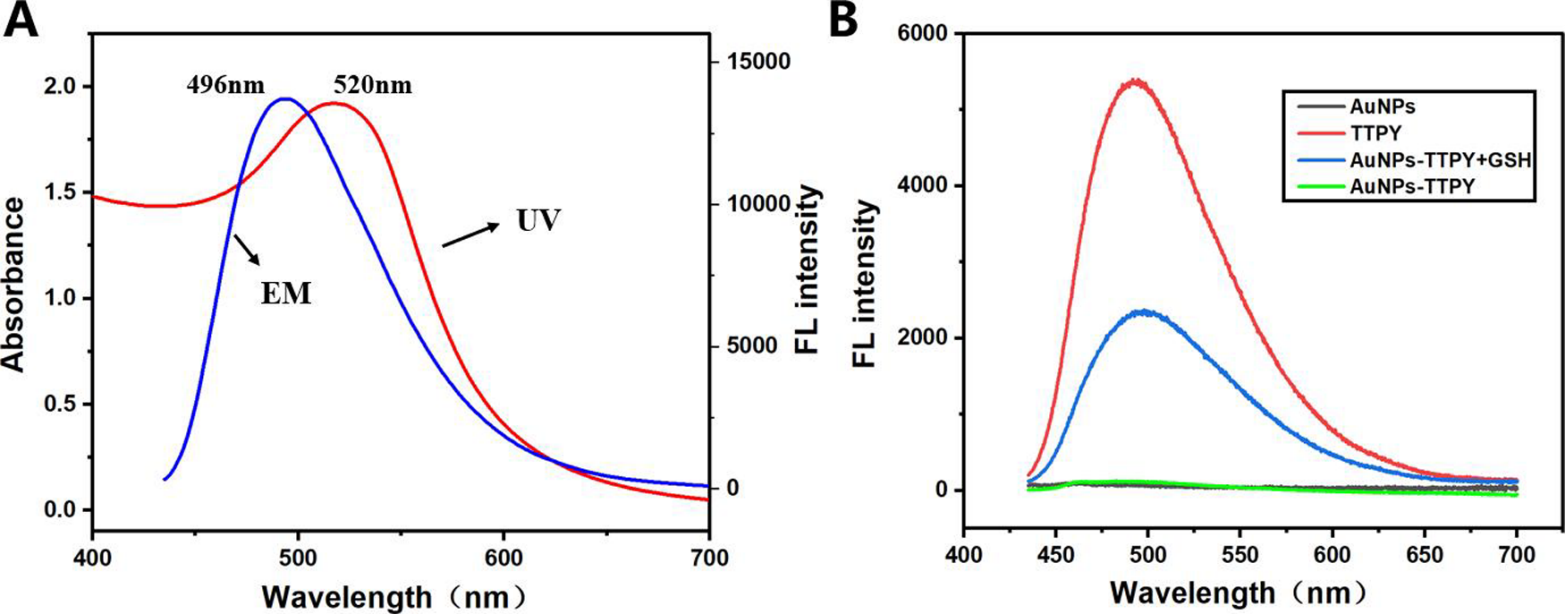
(A) Emission spectra of TTPY and UV–vis absorption spectrum of AuNPs. (B) Emission spectra of AuNP–TTPY responding to 10-μM GSH.

### 3.3 Optimization of Detection Conditions

To achieve high GSH detection sensitivity, we optimized the AuNP concentration, pH, and response time. Initially, the effect of AuNP concentration was investigated. Figure 4A,B show that the AuNP concentration was gradually increased while maintaining a constant TTPY during the fluorescence measurement of the complex. TTPY fluorescence is almost completely quenched at 3.5-nM AuNPs, which was selected as the optimal concentration. The pH effect on the GSH-added AuNP–TTPY complex was examined in the range of 2.5–5.5 (Figure 4C). The results revealed intense and stable fluorescence emission at 496 nm within the pH range of 2.5–4.0, whereas only weak fluorescence was observed at pH > 5.0. To further elucidate the pH-dependent behavior of the sensor, the point of zero charge of the synthesized gold nanoparticles (AuNPs) was determined. As shown in Figure S9, the pH of monodisperse AuNPs lies between 3.0 and 3.5. At low pH (pH < 2.5), the AuNPs surface becomes highly positively charged. Strong electrostatic repulsion exists between the positively charged TTPY and the highly protonated AuNPs surface. This hinders effective approach and adsorption of TTPY onto the AuNPs. Consequently, even upon addition of GSH, the weak initial binding results in minimal fluorescence recovery and a poor signal-to-noise ratio. At high pH (pH > 4.0), the AuNPs surface acquires a strong negative charge. The pronounced electrostatic attraction between positively charged TTPY and negatively charged AuNPs leads to exceptionally tight binding. Although this may initially achieve high quenching efficiency, the excessively strong association impedes efficient displacement of TTPY by GSH. As a result, only negligible fluorescence recovery is observed after GSH addition. This indicates that the sensor operates stably under acidic conditions (pH 2.5–4.0), with optimal performance in this regime. Subsequently, the time response of AuNPs-TTPY complex after GSH addition was studied. Figure 4D shows that upon adding GSH to the sensing system, the fluorescence intensity at 496 nm increases rapidly within 5 min and stabilizes at ∼9 min.

**Figure 4:**
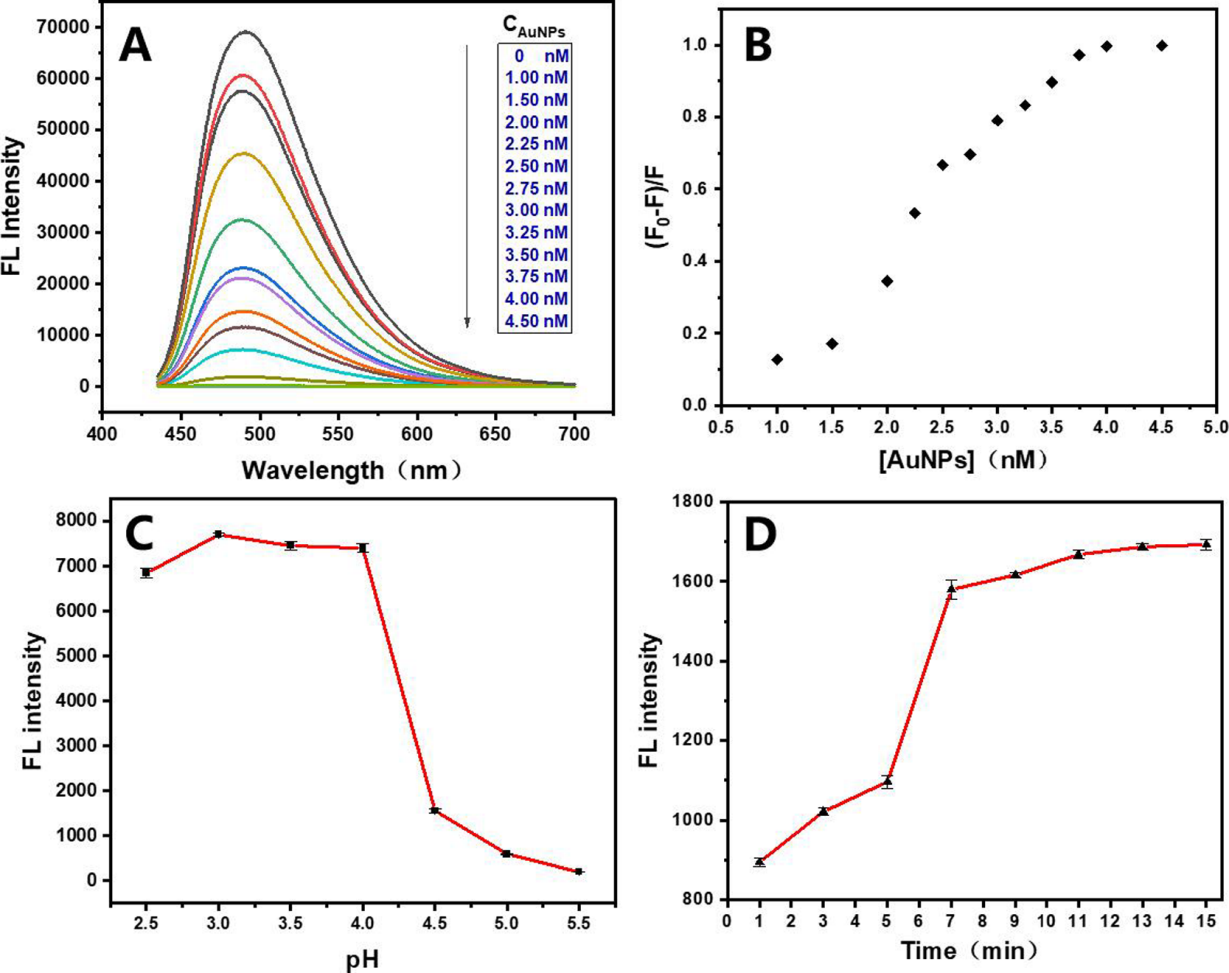
(A) Fluorescence spectra of the TTPY in the presence of different concentrations of AuNPs (0–4.5 nM), (B) effects of AuNP concentration, (C) effects of pH, and (D) effects of incubation time.

Under optimal conditions, fluorescence titration of the AuNP–TTPY complex was performed with varying GSH concentrations. Figure 5A shows that the fluorescence intensity of the AuNP–TTPY complex increases with increasing GSH concentrations. A strong linear correlation is obtained within the GSH concentration range of 0.2–30 μM, with a detection limit of 54 nM (3*s* *k* ^–1^, where *s* is the standard deviation of blank measurements, and *k* is the slope of the linear equation^44^). Compared with other optical nanosensor-based detection methods (Table 1), this approach demonstrates a fast response time and a low detection limit without requiring complex sample pretreatment.^45–48^ This indicates that AuNPs-TTPY serves as a promising probe for GSH detection, enabling quantitative determination of GSH in complex matrices.

**Figure 5:**
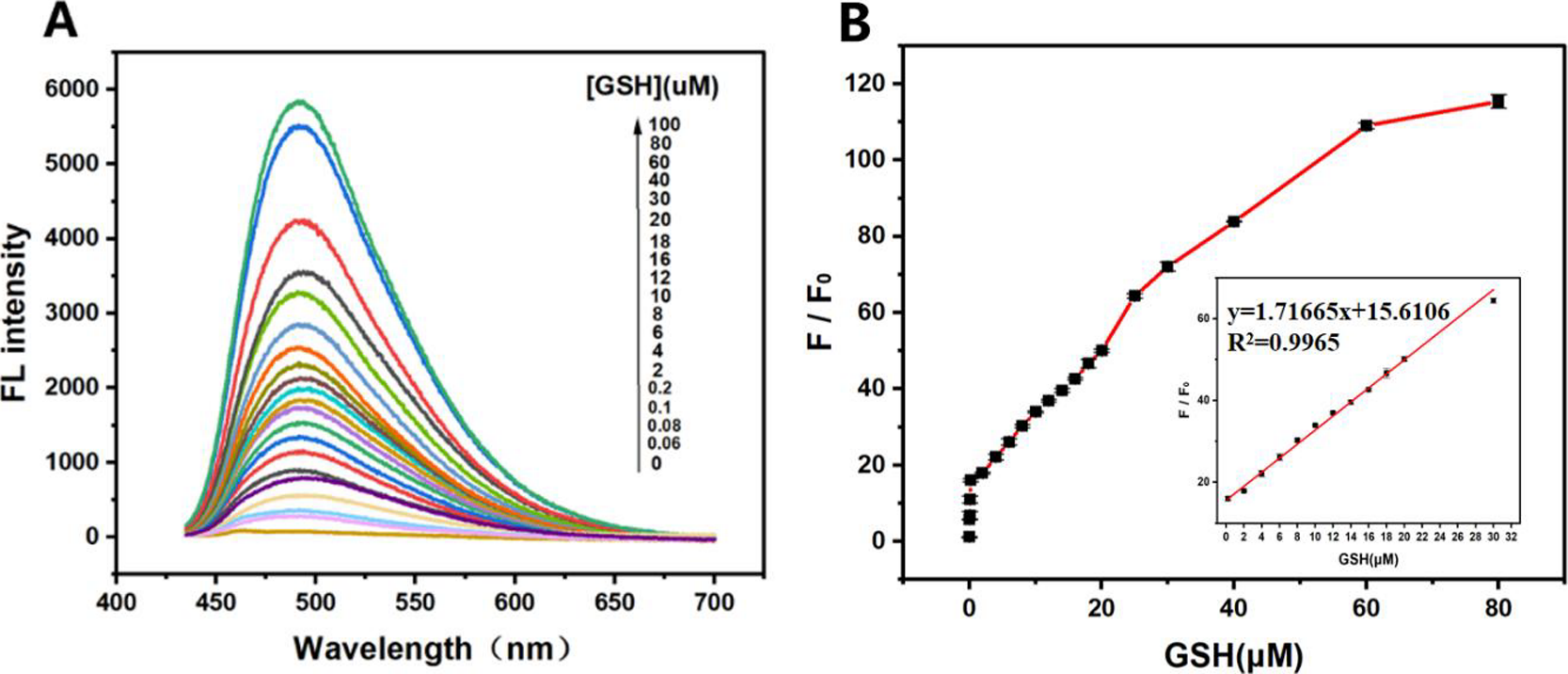
(A) Fluorescence titration of AuNP–TTPY with varying amounts of GSH. (B) Fluorescence intensity of AuNP–TTPY versus the GSH concentration. Each data point is the mean of three measurements. Error bars represent the standard deviation.

**Table 1:**
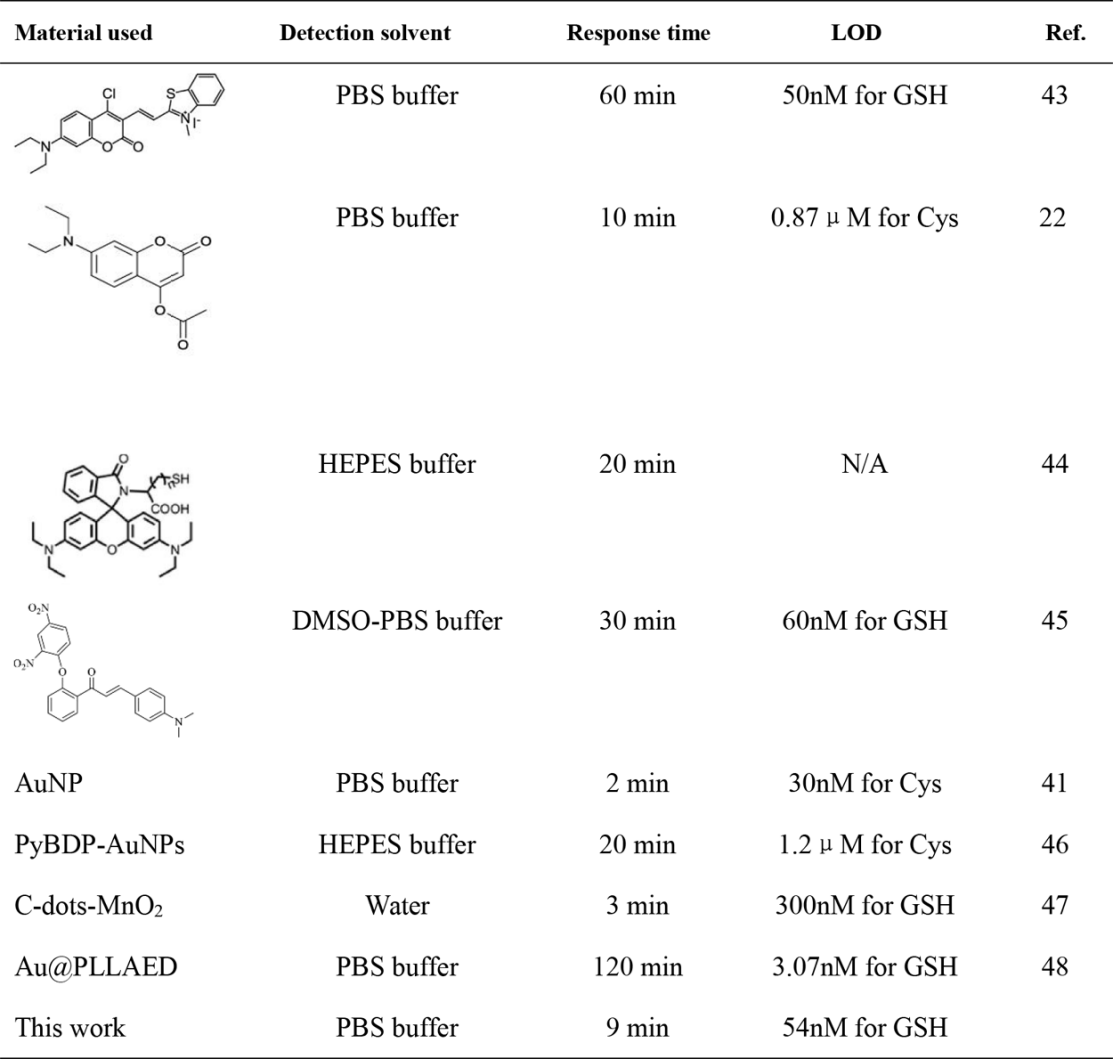
Comparison of Optical Nanosensors for Biothiol Detection

### 3.4 AuNP–TTPY Selectivity Test

Selectivity is a core performance metric for chemical sensors, directly determining their effectiveness and reliability in practical applications. It is crucial to investigate the responses of the AuNP–TTPY fluorescence sensor to common amino acids that might interfere with GSH detection. Figure 6 shows that TTPY fluorescence quenched by AuNPs is largely recovered in the presence of GSH (10 μM). Under the same conditions, no significant changes in fluorescence intensity occur when other amino acids [glycine (Gly), alanine (Ala), valine (Val), leucine (Leu), isoleucine (Ile), phenylalanine (Phe), trypsin (Trp), tyrosine (Tyr), aspartic acid (Asp), histidine (His), asparagine (Asn), glutamic acid (Glu), lysine (Lys), glutamine (Gln), methionine (Met), arginine (Arg), serine (Ser), threonine (Thr), cysteine (Cyst) and proline (Pro)] are present at 10-fold higher concentrations than GSH. This confirms the highly selective sensing capability of AuNP–TTPY. Additionally, a visual fluorescence response of the AuNP–TTPY complex to GSH was captured under a 365 nm UV lamp (Figure 6 inset).

**Figure 6:**
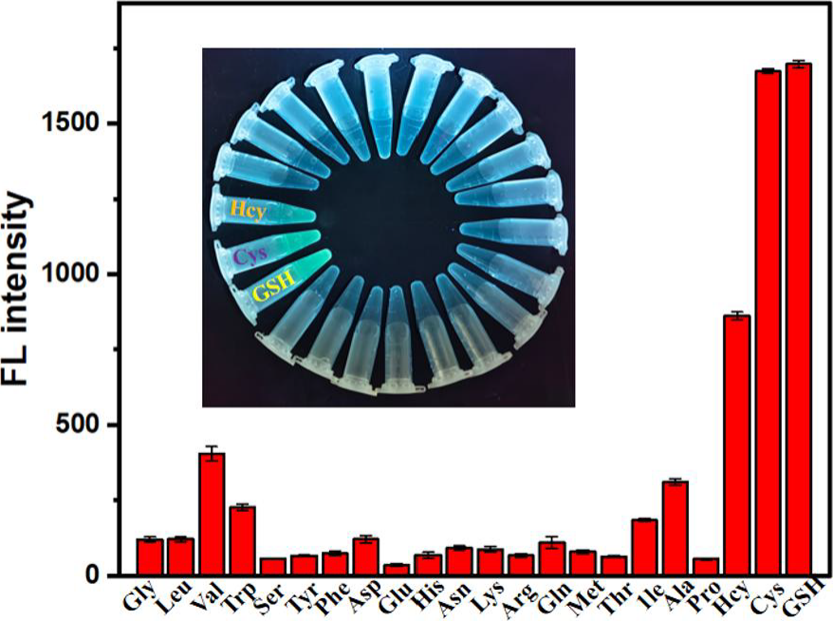
Fluorescence responses of AuNP–TTPY to various amino acids (100 μM), Hcy (10 μM), Cys (10 μM), and GSH (10 μM). Inset: fluorescence image of AuNP–TTPY after 10 min incubation with various amino acids.

### 3.5 Analysis of GSH in Real Samples

The FRET-based AuNP–TTPY sensor was used to detect GSH in three different fruit samples, demonstrating its applicability for real-sample analysis. For detection, pretreated fruit solutions were diluted to the desired concentration range.

Table 2 shows the recovery rates for GSH detection in cucumber (89–101%) with a relative standard deviation (RSD, *n* = 3) of <2.9%, tomato (97–107%; RSD < 2.2%), and pineapple (90–105%; RSD < 2.5%). These results confirm the high accuracy, precision, and reproducibility of the proposed method for rapid GSH detection in real samples, highlighting its practical utility.

**Table 2:** Determination of GSH in Fruit Samples

sample	added (10^–6^ mol L^–1^)	found (10^–6^ mol L^–1^)	recovery (%)	RSD (%, *n* = 3)
pineapple	0	2.4		
	2	4	90	2.5
	4	6.7	105	2.1
tomato	0	4.9		
	2	7.4	107	2.2
	4	8.6	97	1.9
cucumber	0	3.8		
	2	5.9	101	1.5
	4	6.9	89	2.9

## 4 Conclusions

Herein, a rapid turn-on fluorescent sensor for GSH detection was developed based on FRET, using TTPY as the donor and citrate-stabilized AuNPs as the acceptor. The sensor effectively quenched the fluorescence of the fluorophore. In the presence of biothiols, TTPY was displaced from the AuNP surface through competitive thiol–Au coordination, triggering a turn-on fluorescence response. This high-performance sensing platform exhibited exceptional selectivity, water solubility, sensitivity (with a detection limit of 54 nM), and real-time response capability, enabling its successful application for the detection of GSH in food samples. These findings demonstrated that AuNP-based conjugates are a significant versatile platform for biothiol sensing. The methodology validated the feasibility of our approach. It can be used for the future designing of sensing architectures using these functional materials.

## Supplementary Material



## Data Availability

The data that support the findings of this study are available in the Supporting Information of this article.

## Notes

^1^H and ^13^C NMR spectra of synthesized compounds TTPY; material characterization; and optimization of detection conditions and selectivity tests (DOCX)
