# Supplementary material for: Efficient Turn-On Fluorescent Sensor Based on Fluorescent Resonance Energy Transfer between 1,3,6,8-Tetra(4-pyridyl)pyrene and Gold Nanoparticles for Glutathione Detection
Source: ACS Omega. 2025 Oct 16;10(42):49758–65. doi: 10.1021/acsomega.5c05251 (PMC12573021; doi:10.1021/acsomega.5c05251)
Supplement: Supplementary file 1 [file ao5c05251_si_001.docx]

**Efficient Turn-On Fluorescent Sensor Based on Fluorescent Resonance Energy Transfer between 1,3,6,8-Tetra(4-pyridyl)pyrene and Gold Nanoparticles for Glutathione Detection**

Shicong Liu^[a]^, Yuanyuan Zhang^[a]^, Dan Jia^[a]^, Junqiu Liu^[a]^, and Chunxi Hou^[a]^*

1. State Key Laboratory of Supramolecular Structure and Materials, College of Chemistry, Jilin University, 2699 Qianjin Street, Changchun 130012, China

E-mail: [chunxihou@jlu.edu.cn](mailto:chunxihou@jlu.edu.cn)

**Table of contents**

[1.Instrument and methods 2](#_Toc202891456)

[2. Synthesis of TTPY 3](#_Toc202891457)

[3. Characterization of the as-prepared TTPY and AuNPs 5](#_Toc202891458)

[4.TEM images of the synthesized nanomaterial 7](#_Toc202891459)

[5. Optimization of the detection conditions 8](#_Toc202891460)

[6. AuNPs-TTPY selectivity test 9](#_Toc202891461)

1.Instrument and methods

***TEM*:** TEM images were captured with a JEM-2100F transmission electron microscope at an acceleration voltage of 200 kV. The sample was made by 7μL of the stock solution on a 300-mesh, carboncoated copper grid and air-dried.

***DLS*:** 2mL of the stock solution was placed into the glass and the instrument was Malven Instrument zetasizer Nano ZS equipped with a He-Ne laser and an avalanche photodiode detector.

***Fluorescence spectrum*** was obtained using fluorescence spectrophotometer 5301PC equipped with 150 W Xenon lamp, scanning from 435 nm to 700 nm.

***UV-Vis spectroscopy*：**The UV-Vis absorption spectra were measured on a UV-2450 UV-Vis Spectrometer.

***FT-IR*:** The surface functional groups of AuNPs-TTPY were determined by measuring the Fourier Transform Infrared (FTIR) spectra of AuNPs, TTPY, and AuNPs-TTPY solids. The freeze-dried solids were ground thoroughly with dried KBr under infrared light, pressed into pellets, and characterized using a VERTEX 80V Fourier Transform Infrared Spectrometer.

2. Synthesis of TTPY


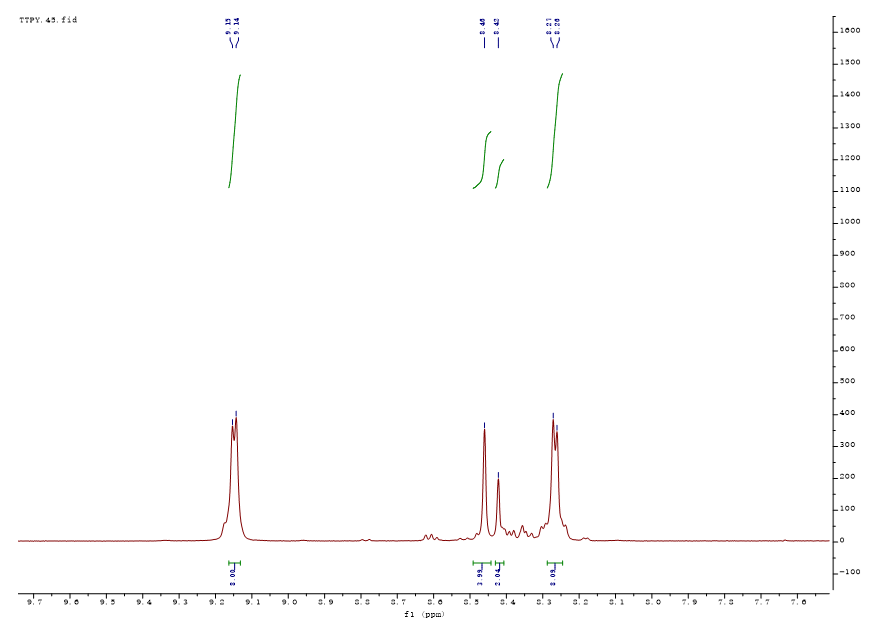
TTPY was synthesized via the Suzuki coupling reaction ^[1]^.
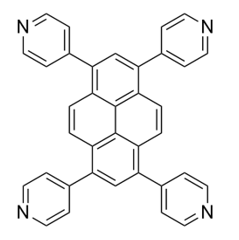
 1,3,6,8-tetrabromopyrene (1.04 g ,2 mmol) was introduced into the reaction flask, accompanied by 983 mg (8 mmol) of 4-pyridylboronic acid, 200 mL of DMF, and 0.42 g of potassium carbonate (K_2_CO_3_). The mixture was degassed by bubbling nitrogen through it for a duration of 30 minutes. Subsequently, Pd(PPh_3_)_4_ (0.138 g ,0.12 mmol) was added, and the solution was further degassed for an additional 10 minutes. The reaction mixture was then heated to 145°C and maintained at this temperature for 48 hours. Upon cooling, the mixture was poured into 1 liter of water and stirred vigorously for 30 minutes to facilitate precipitation. The resulting precipitate was collected by filtration and washed sequentially with 100 mL portions of water, methanol, and dichloromethane. The obtained product is a green solid (851 mg, 1.66mmol, 83%) (500 MHz, Acetic acid-d4): δ(ppm) = 9.10 (d, J 6.0 Hz, 8H), 8.43 (s, 4H), 8.36 (s, 2H), 8.17 (d, J 6.0 Hz, 8H).

**Fig S1.** ^1^H NMR spectral of the TTPY (500 MHz, Acetic acid-d4, 298K).


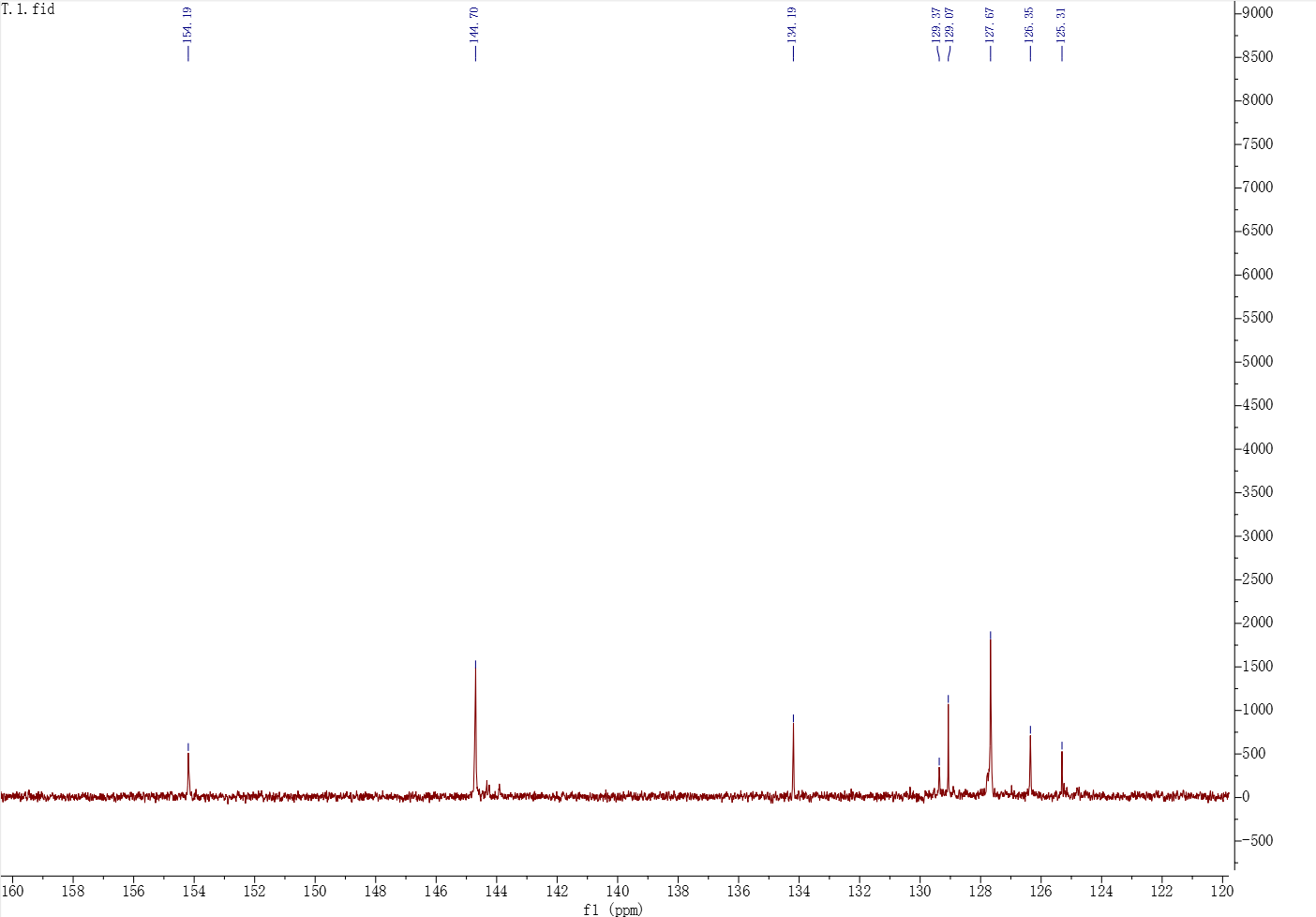


**Fig S2.** ^13^C NMR spectral of the TTPY (600 MHz, Acetic acid-d4, 298K).

3. Characterization of the as-prepared TTPY and AuNPs


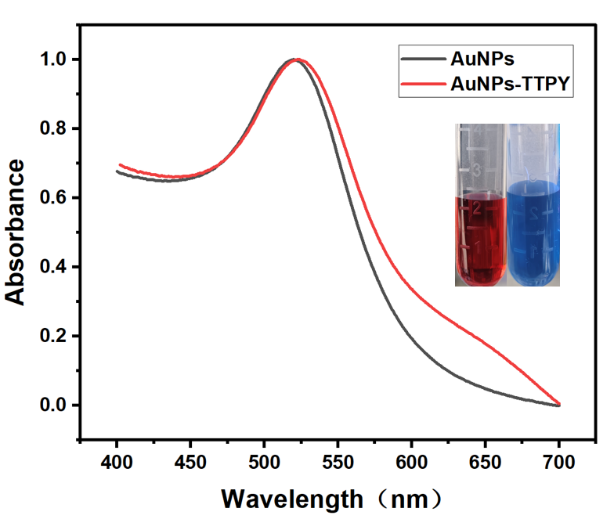
To investigate the successful binding between gold nanoparticles (AuNPs) and 1,3,6,8-tetra(4-pyridyl)pyrene (TTPY), the nanomaterials were characterized by UV-vis spectroscopy and zeta potential measurements. The results revealed a redshift in the absorption peak of gold nanoparticles (AuNPs) after their assembly with 1,3,6,8-tetra(4-pyridyl)pyrene (TTPY), confirming the successful formation of the AuNP-TTPY conjugate.

**Figure S3.** The absorbance spectra of AuNP solution(black) and the AuNP-TTPY solution (red). Inset：Photographs of the AuNPs solution (red) and the AuNPs-TTPY solution (black).

Zeta potential measurements revealed that citrate-capped gold nanoparticles (AuNPs) exhibited a strongly negative surface charge, while 1,3,6,8-tetra(4-pyridyl)pyrene (TTPY) displayed a positive charge in aqueous media. This complementary charge distribution enables robust electrostatic binding between AuNPs and TTPY, forming a stable nanohybrid system.


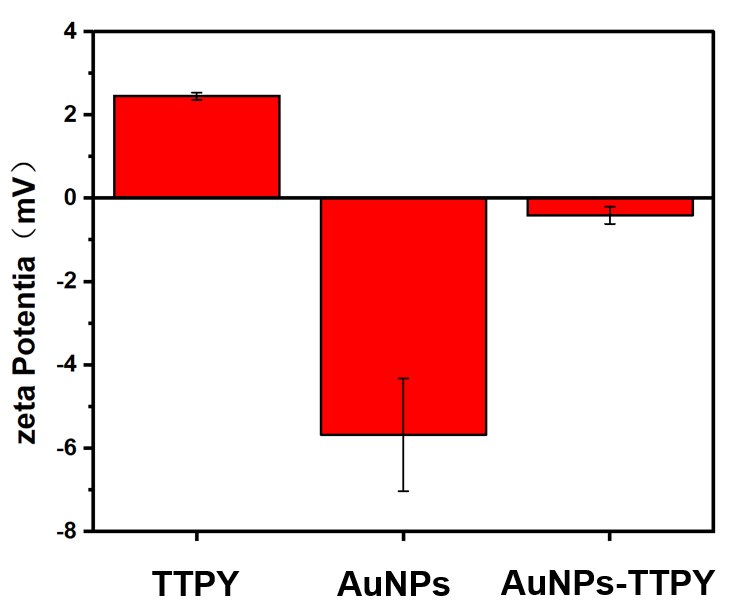


.

**Figure S4.** Zeta potentials measurements of TTPY, AuNPs and AuNPs-TTPY.

To further verify the FRET effect between AuNPs and TTPY, the fluorescence lifetimes and quantum yield of TTPY and AuNPs-TTPY were determined. The results show that the fluorescence lifetime of TTPY is 3.69 ns. Upon conjugation with AuNPs and the occurrence of FRET, the fluorescence lifetime decreases to 3.40 ns. The quantum yield of TTPY is 58.13%. Following fluorescence quenching of TTPY by AuNPs, the quantum yield decreases to 1.87%.


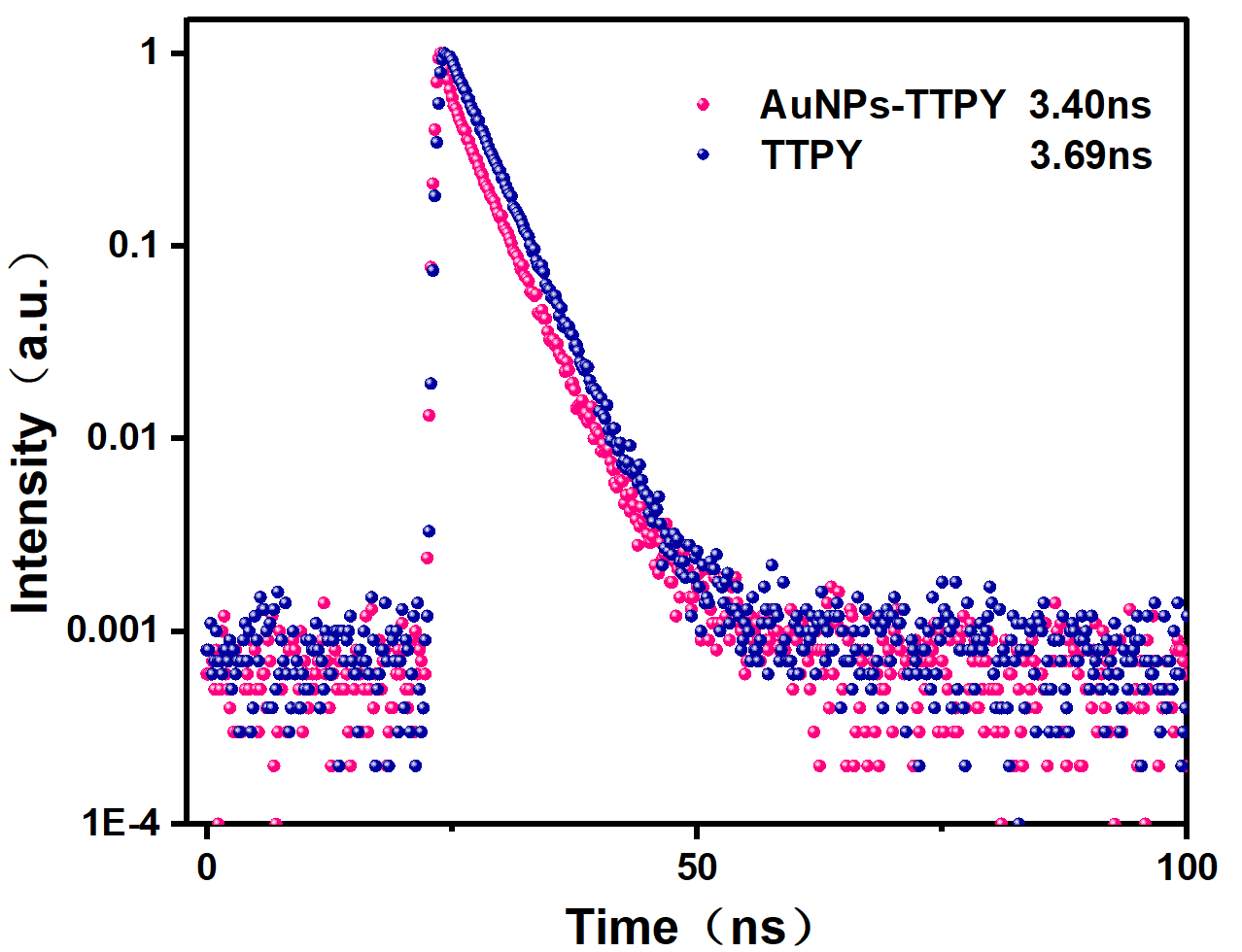


**Figure S5.** The blue curve represents the fluorescence lifetime of TTPY, and the pink curve represents the fluorescence lifetime of AuNPs-TTPY.


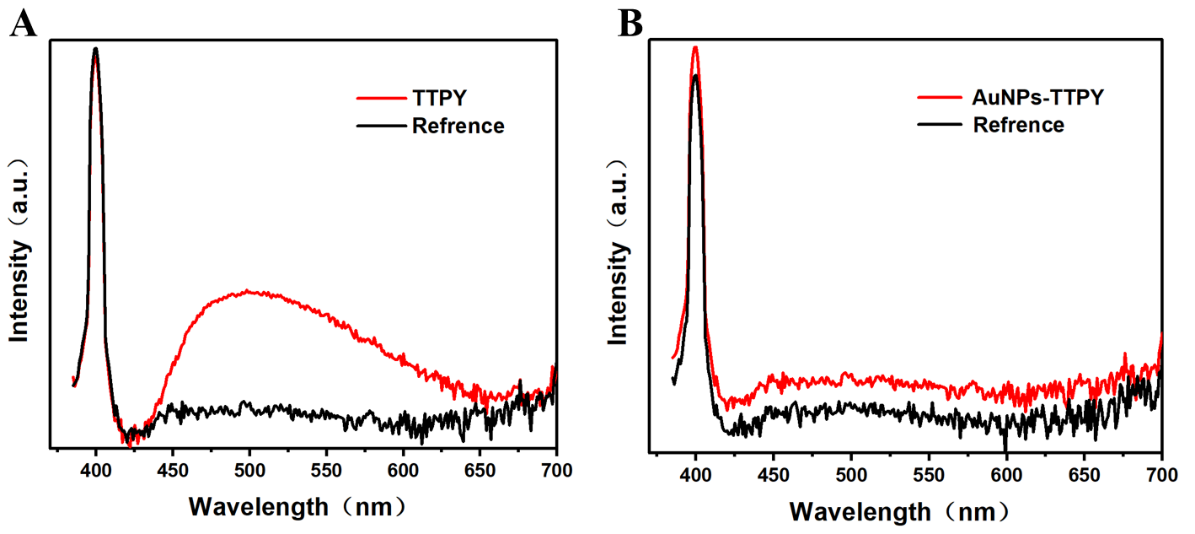
**Figure S6.** (A) Quantum yield spectrum of TTPY, (B) Quantum yield spectrum of AuNPs-TTPY.

4.TEM images of the synthesized nanomaterial


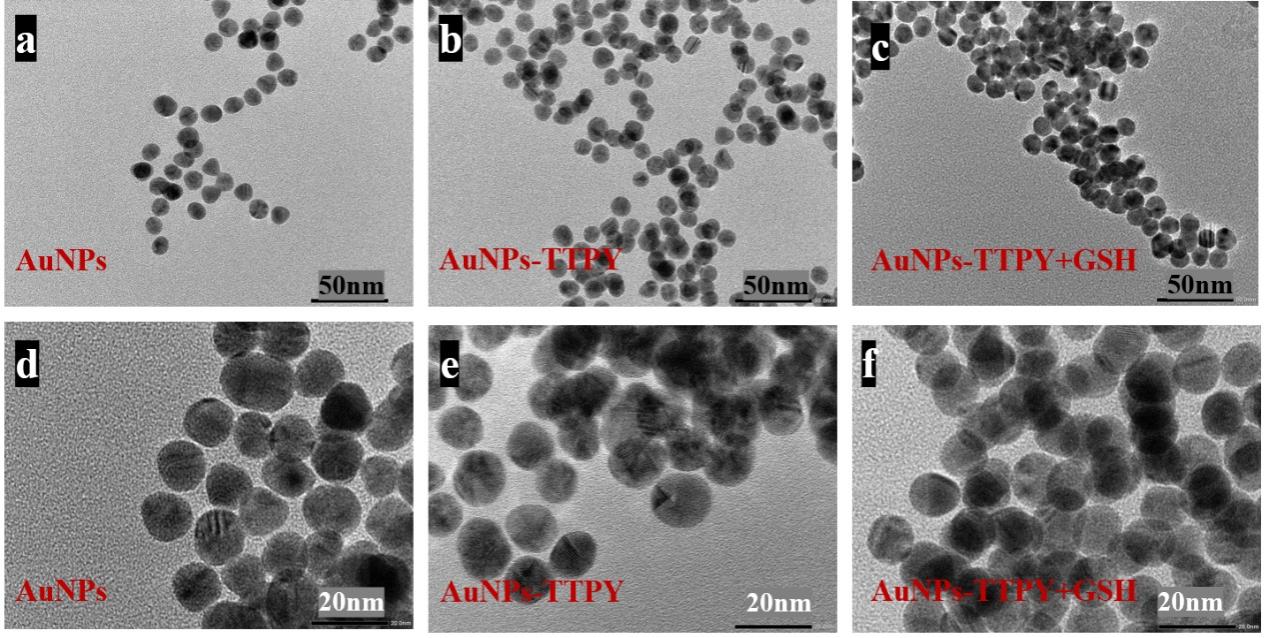
To investigate the morphology of AuNPs and the effects of TTPY and GSH on the morphology of AuNPs, the nano-materials were subjected to TEM testing. The TEM images revealed that the addition of TTPY and GSH did not significantly alter the morphology or aggregation state of the AuNPs. All three systems (monodisperse AuNPs, AuNPs-TTPY, and AuNPs-TTPY-GSH) exhibited similar size distributions. The monodisperse AuNPs exhibited an average diameter of approximately 13.8 nm. Upon the addition of TTPY, the average size of the AuNPs-TTPY conjugate slightly increased to 14.1 nm, indicating the formation of a thin molecular layer of TTPY on the AuNP surface. Notably, the introduction of glutathione (GSH) did not induce any significant changes in the average size of the AuNPs (14.2 nm), confirming that GSH-triggered ligand exchange occurs without disrupting the structural integrity of the AuNPs.

**Figure S7.** TEM images:(a) (d) monodispersed AuNPs, (b) (e) in the presence of TTPY, (c) (f) in the presence of TTPY and GSH.

5. Optimization of the detection conditions

To achieve high sensitivity for GSH detection, we optimized experimental conditions including pH value and response time. The results revealed intense and stable fluorescence emission at 496 nm within the pH range of 2.5–4.0, whereas only weak fluorescence was observed at pH >5. This indicates that the sensor operates stably under acidic conditions (pH 2.5–4.0), with optimal performance in this regime. Upon adding GSH to the sensing system, the fluorescence intensity at 496 nm increased rapidly within 5 minutes. The fluorescence continued to increase and stabilized at about 9 minutes.


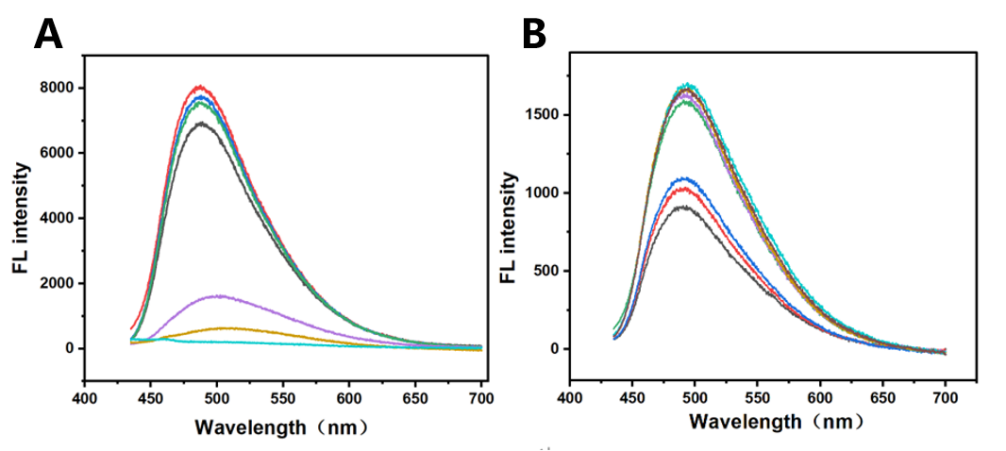


**Figure S8.** (A) pH-dependent (2.5-5.5) and (B) time-dependent (0-15 min) fluorescence spectra of AuNPs-TTPY-GSH.

To further elucidate the pH-dependent behavior of the sensor, the point of zero charge of the synthesized gold nanoparticles (AuNPs) was determined. As shown in Figure S9, the pHzpc of monodisperse AuNPs lies between 3.0 and 3.5.


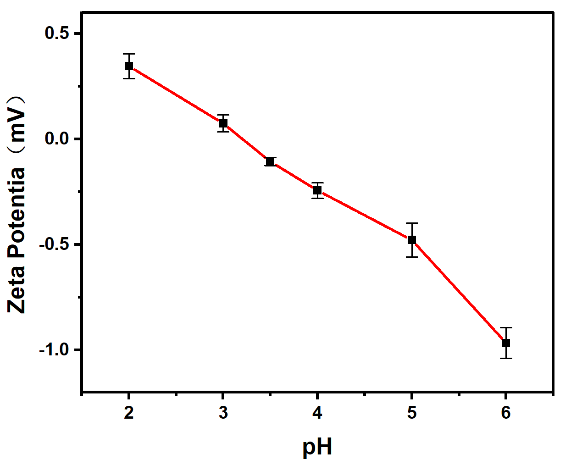


**Figure S9.** Zeta Potential of AuNPs at Different pH Values.

6.AuNPs-TTPY selectivity test

To investigate the responses of the AuNPs-TTPY fluorescence sensor to common amino acids that may interfere with GSH detection, the selectivity test was conducted.

As shown in Figure S6, significant fluorescence recovery of AuNPs-quenched TTPY occurred in the presence of thiol compounds (10 μM each: GSH, Cys, Hcy). Under identical conditions, no appreciable change in fluorescence intensity was observed even when concentrations of other amino acids (namely Gly, Leu, Val, Trp, Ser, Tyr, Phe, Asp, Glu, His, Asn, Lys, Gln, Met, Arg, Ala, Ile, Thr, Pro) were elevated to 10-fold that of the thiol analytes.


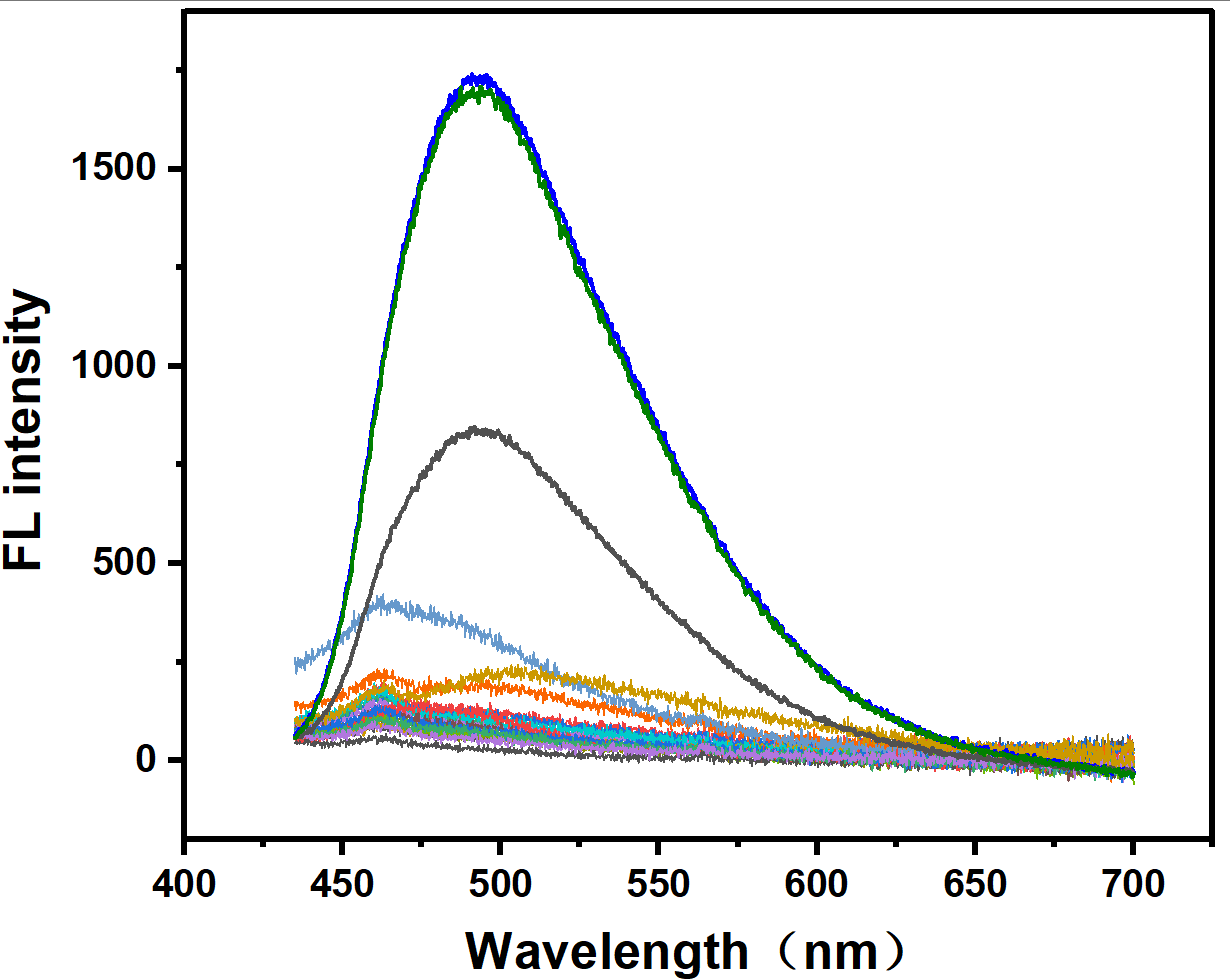


**Figure S10.** Fluorescence spectra of AuNPs-TTPY to various amino acids（100 μM） and GSH（10 μM）.

References

[1]Y Yan, R.H.; Cui, W.R.; Jiang, W.; Huang, J.; Liang, R.P.; Qiu, J.D.; Rationally designed pyridinium cationic polymeric network for effective TcO4−/ReO4− remediation. *Chemical Engineering Science*. **2023**, *268*, No.118403.
